# Cytoprotective Action of Sodium Fumarate in an *in vitro* Model of Hypoxia Using Sodium Dithionite

**DOI:** 10.17691/stm2025.17.1.09

**Published:** 2025-02-28

**Authors:** A.Yu. Vinokurov, S.V. Popov, D.Yu. Belyakov, D.Yu. Popov, A.S. Nikulin, V.D. Zakrzhevskaya, R.G. Guseinov, K.V. Sivak, A.V. Dunaev, E.V. Potapova, A.Yu. Abramov

**Affiliations:** PhD, Senior Researcher, Research and Development Center of Biomedical Photonics; Orel State University, 95 Komsomolskaya St., Orel, 302026, Russia; MD, DSc, Professor, Chief Physician; Clinical Hospital of St. Luke, 46 letter A, Chugunnaya St., Saint Petersburg, 194044, Russia; Head of the City Center for Endoscopic Urology and New Technologies; Clinical Hospital of St. Luke, 46 letter A, Chugunnaya St., Saint Petersburg, 194044, Russia; Professor, Department of Urology; Military Medical Academy, 6 letter Zh, Akademika Lebedeva St., Saint Petersburg, 194044, Russia; Head of the Department of Surgery and Urology; Saint Petersburg Medical and Social Institute, 72 letter A, Kondratyevsky Prospekt, Saint Petersburg, 195271, Russia; Laboratory Assistant, Laboratory of Cell Physiology and Pathology, Research and Development Center of Biomedical Photonics; Orel State University, 95 Komsomolskaya St., Orel, 302026, Russia; Research Intern, Laboratory of Cell Physiology and Pathology, Research and Development Center of Biomedical Photonics; Orel State University, 95 Komsomolskaya St., Orel, 302026, Russia; Student; Novosibirsk State University, 1 Pirogova St., Novosibirsk, 630090, Russia; Laboratory Assistant, Laboratory of Cell Physiology and Pathology, Research and Development Center of Biomedical Photonics; Orel State University, 95 Komsomolskaya St., Orel, 302026, Russia; MD, PhD, Deputy Chief Physician for Research; Clinical Hospital of St. Luke, 46 letter A, Chugunnaya St., Saint Petersburg, 194044, Russia; Senior Lecturer, Department of Surgery and Urology; Saint Petersburg Medical and Social Institute, 72 letter A, Kondratyevsky Prospekt, Saint Petersburg, 195271, Russia; Assistant Professor, Department of Hospital Surgery; Saint Petersburg State University, 7/9 Universitetskaya naberezhnaya St., Saint Petersburg, 199034, Russia; DSc, Leading Researcher; Clinical Hospital of St. Luke, 46 letter A, Chugunnaya St., Saint Petersburg, 194044, Russia; Head of the Department of Preclinical Studies of Medications; A.A. Smorodintsev Research Institute of Influenza of the Ministry of Healthcare of the Russian Federation, 15/17 Professora Popova St., Saint Petersburg, 197376, Russia; DSc, Leading Researcher, Research and Development Center of Biomedical Photonics; Orel State University, 95 Komsomolskaya St., Orel, 302026, Russia; PhD, Senior Researcher, Research and Development Center of Biomedical Photonics; Orel State University, 95 Komsomolskaya St., Orel, 302026, Russia; DSc, Head of the Laboratory of Cell Physiology and Pathology, Research and Development Center of Biomedical Photonics; Orel State University, 95 Komsomolskaya St., Orel, 302026, Russia; Professor; UCL Queen Square Institute of Neurology, Queen Square, London WC1N 3BG, United Kingdom

**Keywords:** hypoxia, cell culture, sodium fumarate, sodium dithionite, apoptosis

## Abstract

Hypoxia is a part of many pathological and some physiological processes. It also occurs as a result of surgical techniques associated with limiting the blood supply to the operated organs and tissues. Hypoxia leads to a significant decrease in the ability of cells to implement energy-dependent processes due to a reduced contribution of mitochondria to the synthesis of adenosine triphosphate (ATP). In order to protect cells and increase the time of surgery, infusion of a solution of sodium fumarate for several days before the surgical procedure is suggested. However, the mechanism of the observed protective effect is still a subject of discussion.

**The aim of the research was to study** the mechanism of the sodium fumarate cytoprotective effect on renal epithelial cells in acute hypoxia modeling *in vitro* by reducing oxygen in the medium using sodium dithionite.

**Materials and Methods:**

The study was conducted using the MDCK renal epithelial cell line with sodium dithionite at a concentration of 5 mM to create hypoxic conditions. The parameters of cellular metabolism (including the value of mitochondrial membrane potential, the state of mitochondrial NADH and FAD, the content of Ca^2+^ and Mg^2+^ and the pH level in the cytosol, the rate of glucose absorption by cells, and cell death) were assessed by means of confocal and wide-field fluorescence microscopy. The concentration of dissolved oxygen was established using the polarographic method with a Clark electrode.

**Results:**

It was demonstrated that the use of sodium dithionite allows modeling acute hypoxia *in vitro* with a rapid decrease in the oxygen concentration in the cell incubation medium, which resulted in a change in mitochondrial function and the apoptosis progression. At that, sodium fumarate reduces the level of cell death, which is associated not with the restoration of the ATP-producing ability of mitochondria, but rather with an increase in the contribution of alternative sources of high-energy compounds.

**Conclusion:**

At the cellular level, using an optimized hypoxia model, the study revealed the mechanism of the protective role of sodium fumarate, which explained the antihypoxant effectiveness in assisted ischemia of organs and tissues.

## Introduction

Hypoxia, characterized by a decrease in the oxygen partial pressure in organs and tissues, is a significant part of many pathological and some of physiological processes [1, 2]. Moreover, hypoxia may occur when using organ-preserving techniques during surgery, for example, during kidney resection, which is used to remove small localized tumors being typical of T1a, T1b, and T2 developmental stages [3, 4]. In this case, blood circulation blocking in the resected organ by cold or warm ischemia can be used in order to better control the boundaries of tumor growth by visualizing macroscopic differences between the malignant tumor and healthy tissue, reduction of blood loss and maintenance of hemostasis during surgery [5, 6]. The hypoxia resulting from the blood flow arrest and, consequently, insufficiency of biological oxidation and energy imbalance ultimately lead to cell death [7]. Negative consequences are also observed as a result of reoxygenation after restoration of normal oxygen concentration in the tissue [6]. Although changes in the kidney tissue are seen even in the first minutes of the procedure [8], the majority of researchers mention 25–30 min as the period after which these changes become critical and irreversible [3, 5, 8–11]. Probably, this is due to the fact that the main cellular mechanism that maintains the cell energy balance in case of ischemia is anaerobic glycolysis, but its capacities in kidney cells are exhausted after 15–20 min of ischemia [12]. In general, in surgery lasting over 30 min, the use of cold ischemia is recommended, the relatively safe duration of which can be up to one hour [5], or pharmacological nephroprotection, including with the help of antihypoxants [13].

Antioxidants [14, 15], mannitol [16, 17], and substrates of the Krebs cycle and mitochondrial electron transport chain (ETC) complexes (for instance, fumarate) [6], can be used as antihypoxants to protect the kidneys during warm ischemia. Preclinical and clinical studies have demonstrated that intravenous administration of sodium fumarate (Na-fumarate) in advance significantly improves renal function immediately after the procedure, as well as in the longer perspective.

It should be noted that the mechanism of action of potential antihypoxants both during hypoxia followed by reoxygenation, as well as the possibility of increasing the acceptable duration of blood flow restriction in the kidney are still poorly understood. The following processes can be mentioned as suggested explanations for the protective effect: ATP synthesis under anaerobic conditions due to substrate phosphorylation [18, 19] and due to the ability of ETC complex I to transfer protons into the intermembrane space for subsequent operation of F1–F0–ATP synthase [18–20]; as well as activation of intracellular processes associated with stabilization of the transcription factor HIF1α [21–28]. A wide range of possible protective mechanisms and the complexity of ischemic damage to the renal parenchyma, the understanding of which is necessary to substantiate nephroprotective therapy [17], require studies based on relevant models, starting from the cellular level.

Approaches to modeling hypoxia *in vitro* can be conditionally divided into two groups: 1) indirect, inhibiting processes that involve oxygen or reactions accompanying hypoxia progression; 2) direct, associated with ensuring a minimum oxygen content in the gas atmosphere and/or in the incubation medium. The use of cobalt chloride [29, 30], which blocks HIF1α degradation and, thus, activates a cascade of hypoxiainduced processes, is an example of the first group of approaches. One can also use cyanides and azides, inhibitors of mitochondrial ETC complex IV, where oxygen is reduced during respiration [31]. Common disadvantages of such compounds include their toxicity [32, 33] and the lack of impact on other processes related to availability of oxygen in the system, which may raise a doubt in the hypoxia model quality. To implement the second group of approaches, special incubators or chambers with a controlled gas ability can be used, providing the ability to reduce the dissolved oxygen content to 1–2% [29, 32, 34]. Despite significant clear advantages, this approach has disadvantages that is the need to use special equipment, a gas mixture, as well as the difficulty of maintaining the gas composition when additional manipulations with cells are expected. A suggested method that allows eliminating some of the said issues is using a high layer of the medium, providing a diffusion-limited low rate of oxygen supply to the adhesive cell layer [27, 32]. However, it is obvious that the required level of hypoxia in the latter case can be achieved after a rather long waiting time. The known methods of oxygen removal from the cell incubation medium include the use of enzymes (for example, simultaneous introduction of glucose oxidase and catalase) [32, 35] or sodium dithionite (Na_2_S_2_O_4_) [34, 36]. At that, the concentration of gluconolactone formed during the enzymatic reaction can significantly exceed the level of cytotoxicity [37]. The approach based on the introduction of Na_2_S_2_O_4_ into the medium is relatively simple and leads to an abrupt decrease in the oxygen content. This makes Na_2_S_2_O_4_ very convenient for modeling acute hypoxia that happens after complete loss of blood circulation [38].

Along with the choice of an approach to create hypoxic conditions, one must determine a method to assess changes in the cell under oxygen deficiency. Experimental determination of the level of proteins forming the HIF1 complex is complicated in screening studies. Instead, one can consider identifying the progression of cell death through both apoptosis and necrosis [39]. However, this requires clarification for a specific model, especially with the account to current ideas about the mechanisms of cell death pathways progression [40].

In this regard, the **research was aimed** to study the mechanism of cytoprotective effect of sodium fumarate on renal epithelial cells in modeling acute hypoxia *in vitro* by reducing oxygen in the medium with sodium dithionite.

## Materials and Methods

### Cell culture

All stages of the study were conducted using the Madin–Derby canine kidney cells (MDCK) cell line, which was cultured in a DMEM-based medium (PanEco, Russia) containing 10% FBS (Biological Industries, Israel), 1 mM sodium pyruvate (Gibco, USA), 2 mM L-alanyl-L-glutamine (Gibco, USA), 100 μg/ml streptomycin and 100 U/ml penicillin (Gibco, USA). Cell cultures were incubated at 37°C in a humidified atmosphere containing 5% CO_2_ and 95% air. The density of the cell monolayer during the research was at least 60%.

### Reagents

To simulate hypoxic situation by reducing dissolved oxygen, the authors used Na_2_S_2_O_4_ (PanReac AppliChem, Germany). Oxygen was removed by achieving a Na_2_S_2_O_4_ concentration of 5 mmol/L by adding a freshly prepared stock solution. Na-fumarate was sourced from the pharmacopoeial preparation Confumin (Medpolymer, Russia), being a 15% solution of Na-fumarate void of impurities. For cell incubation, Confumin was added to the medium until a concentration of 5 mmol/L was achieved.

### Confocal microscopy analysis

Fluorescence measurements were performed using an LSM 900 confocal microscope (Carl Zeiss, Germany). The illumination intensity was minimized (0.1–0.2% of the maximum laser power) to avoid photobleaching.

### Assessment of mitochondrial membrane potential (ΔΨm)

Comparative assessment of the value and analysis of the ΔΨm maintenance mechanism was conducted using the cationic fluorescent tetramethylrhodamine probe (TMRM; Invitrogen, USA) (excitation/fluorescence maxima ~553/578 nm). The accumulation of the probe in mitochondria is determined by the magnitude of the negative charge on the inner mitochondrial membrane. Before the examination, the cells were incubated in a 25 nM probe solution for 45 min [41]. The ΔΨm value was assessed based on the fluorescence intensity. For that, experiments were performed in the layer-by-layer scanning mode of each field of view, followed by isolation of all cells separately and determination of the maximum signal value for each cell. In order to analyze the mechanism of ΔΨm maintaining within one field of view, changes in fluorescence intensity were recorded after sequential addition of complex V inhibitors (oligomycin A, 2 μg/ml) and ETC complex I (rotenone, 2 μM), as well as the FCCP mitochondrial uncoupler (2 μM) [42]. When processing the results of experiments on analysis the ΔΨm maintaining mechanism, normalization of TMRM fluorescence intensity was conducted, where the initial signal level was equal to 1, and the final signal, corresponding to complete depolarization of the mitochondria, to 0.

### Assessment of apoptosis and necrosis

Assessment of necrosis in cell culture was performed using fluorescent probes Hoechst 33342 (Invitrogen, USA) and propidium iodide (Invitrogen, USA). Cells were incubated with 5 μM Hoechst 33342 (excitation/ fluorescence maxima ~350/481 nm) and 5 μM propidium iodide (excitation/fluorescence maxima ~535/617 nm) for 30 min at 37°C.

To assess apoptosis, the NucView^®^ 488 Caspase-3 substrate (NucView, Biotium, USA) (488 nm excitation laser with emission above 515 nm) was used in combination with Hoechst 33342 to detect caspase-3/7 activation and visualize morphological changes in the nucleus during apoptosis. Cells were incubated with 5 μM NucView and 5 μM Hoechst 33342 for 30 min at room temperature [43].

### Assessment of duration of the transmembrane Ca^2+^ gradient maintenance by cells under conditions of ATP synthesis blocking

Comparative assessment of the cells ability to implement energyconsuming processes based on the initial level of ATP was performed using the Mag-Fura-2, AM ratiometric fluorescent probe (Invitrogen, USA), which has a high affinity for Mg^2+^ and a low affinity for Ca^2+^. In both cases, the formation of a probe complex with metals results in a shift in the fluorescence excitation peak from 380 to 340 nm, at that maintaining the maximum fluorescence intensity close to 530 nm. The ratio of fluorescence intensity upon excitation by wavelengths of 340 and 380 nm (F_340_/F_380_) was used as an analytical signal, which characterized the change in the ATP state in cells. When the ATP synthesis pathways are blocked, the growth of this parameter corresponds to the consumption of ATP and the release of Mg^2+^. When ATP stock is completely depleted, cells lose the ability to maintain the difference in Ca^2+^ concentrations relative to the cytoplasmic membrane, which is characterized by a significant increase in the signal [42]. The analysis was performed using a widefield fluorescence microscope based on Olympus IX73P1F (Olympus Corporation, Japan) and a Cairn fluorescence excitation and recording unit (Cairn Research Ltd., UK) with a fluorite immersion objective 40×, using two wavelengths of excitation radiation of a xenon arc lamp — 340 and 380 nm. Before the study, the cells were incubated in a 3 μM probe solution.

### Study of nicotinamide adenine dinucleotide (NADH) autofluorescence

In order to assess the state of ETC complex I under model hypoxia conditions, as well as the impact of Na-fumarate, the autofluorescence level was examined using a wide-field fluorescence microscope with a 40× fluorite immersion objective and exciting radiation (wavelength 360 nm). Fluorescence was recorded in the wavelength range of 430–480 nm.

### Study of flavin adenine dinucleotide (FAD) autofluorescence

Under the influence of succinate dehydrogenase, there is a reversible change in the state of the coenzyme (transition from FAD to FADH_2_ and vice versa), which allows using FAD autofluorescence as a tool to assess the function of ETC complex II. The examinations were conducted using an LSM 900 microscope (Carl Zeiss, Germany) equipped with a laser having a wavelength of 488 nm and recording fluorescence in the range of 490–600 nm.

### Study of the kinetics of glucose absorption by cells

To solve this issue, a fluorescent glucose analog, 2-NBDG (Invitrogen, USA), was used at a concentration of 10 μM. The examinations were conducted using an LSM 900 microscope (Carl Zeiss, Germany) (excitation wavelength — 488 nm, fluorescence recording — over 490 nm) in the following sequence: first by recording the cells autofluorescence, and second — by recording the signal caused by the penetration of the probe into the cells [44].

### Determination of the cytoplasm pH level

The pH in the cytoplasm was assessed using a ratiometric BCECF probe (Invitrogen, USA) on a wide-field fluorescence microscope using a fluorite immersion objective 40× and two excitation wavelengths — 430 and 495 nm. The cells were incubated for 30 min in a 5 μM probe solution, and then were washed and the fluorescence level was identified. The F_495_/F_430_ ratio was used as an analytical signal. To construct a calibration dependence of F_495_/ F_430_ on pH level, the probe signal was titrated in each experiment by sequentially adding potassium-containing buffer solutions with pH 7.8; 7.4 and 6.7, containing 10 μM nigericin [45].

### Data analysis and statistics

Microscopic analysis experiments were conducted at least three times; at least 5 fields of view were used for analysis of individual images. Statistical analysis was performed using the OriginPro software (OriginLab Corp., USA). Differences between the compared groups were assessed using the nonparametric statistical Mann–Whitney U-test. Data were presented as Me [Q1; Q3], and the following notations were used: N — number of experiments; n — number of cells or images.

## Results

*Use of Na_2_S_2_O_4_ results in a time-limited decrease in the oxygen concentration in the solution to hypoxic levels.*

**Figure 1.**
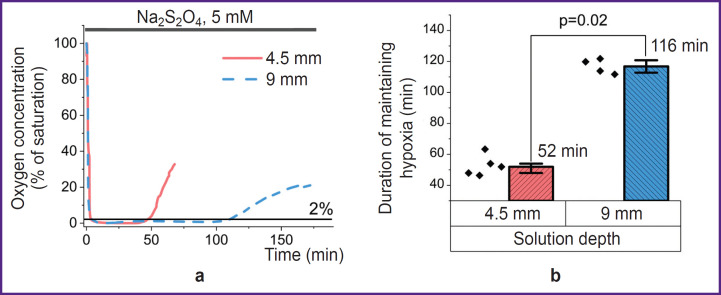
Application of Na_2_S_2_O_4_ ensures a low concentration of dissolved oxygen, the duration of which depends on the solution depth Plots of polarographic determination of changes in oxygen in the medium upon the introduction of Na_2_S_2_O_4_ (a) and the dependence of the duration of maintaining hypoxia on the height of the liquid column (b) (N=5)

In order to assess the conditions for using the Na_2_S_2_O_4_-based method for modeling acute hypoxia, a stock 1 M Na_2_S_2_O_4_ solution was applied, which — when added to the medium — resulted in a concentration of 5 mM. This concentration facilitated hypoxia progression without exerting any other toxic effect on the cells or changing the pH of the solution [34, 41]. Hanks’ balanced salt solution was used as a physiologically acceptable medium in this and subsequent experiments. Examinations using an Oxytherm+ System oximeter (Hansatech Instruments Ltd, UK) established that the addition of a reducing agent sharply decreased the oxygen content in the solution (Figure 1 (a)) to a level of 1–2% of saturation. Due to its low stability, the oxygen-binding ability of Na_2_S_2_O_4_ was time-limited [34]. In the case of a 9 mm solution, the oxygen concentration was kept at the level of less than 1% of the maximum solubility for about 100 min, and then was followed by a rapid increase to a level that prevents stating any hypoxia in the system. A two-fold decrease in the solution layer thickness led to a comparable decrease in the period of dithionite action (Figure 1 (b)). Here, even minor mixing, which apparently affected the rate of gas diffusion, sharply reduced this period. All the above-mentioned must be taken into account when setting up experiments and, in the case of their long duration, researchers must change the solution containing Na_2_S_2_O_4_ or exclude contact of the model system with atmospheric air. In further experiments, the cells were incubated in a medium with a layer thickness of 4.5 mm to create short-term hypoxia. Longer experiments were carried out in a cell with cover glasses used as the bottom and top covers (the lower cover glass contained a monolayer of the adhesive culture of the studied cells).

*Removal of oxygen from the solution caused by Na_2_S_2_O_4_ results in a decrease in cell viability comparable to complete blocking of ATP synthesis pathways.*

**Figure 2.**
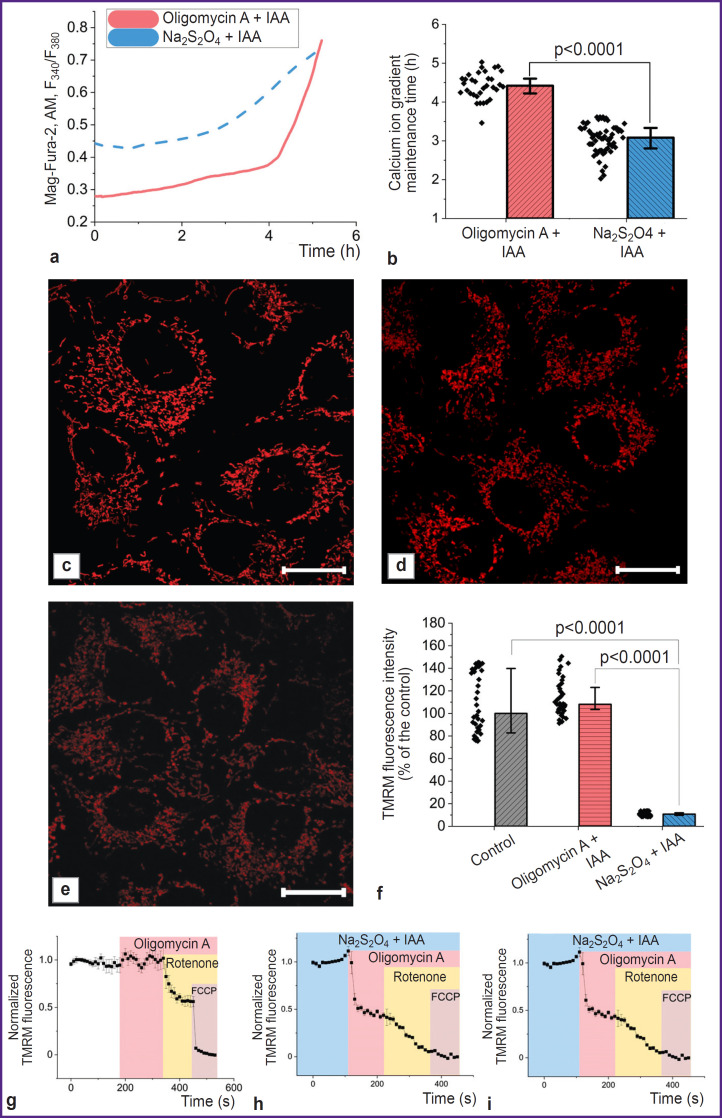
Oxygen reduction with Na2S2O4 results in complete blocking of OXPHOS Plots of changes in the cytosolic concentration of Ca^2+^ and Mg^2+^ (a) and the results of statistical analysis of the duration of maintained concentration gradient of calcium ions relative to the cytoplasmic membrane in two options of OXPHOS blocking (b); confocal images of cells loaded with TMRM in control (c); incubated with oligomycin A and iodoacetic acid (IAA) for 30 min (d); incubated with Na2S2O4 and IAA for 30 min (e); results of processing the TMRM fluorescence intensity data (f) for two OXPHOS blocking options compared to the control; plots reflecting the mechanism of ΔΨm value maintaining in the control experiment (g); as well as for two OXPHOS blocking options (h), (i). Bar is 20 μm

In order to monitor changes in the state of subjected to hypoxia cells over time, the authors assessed the kinetics of the increase in intracellular Mg^2+^ and Ca^2+^ concentrations resulting from ATP hydrolysis and the Ca^2+^ entering the cytoplasm from the medium, respectively, under conditions of ATP synthesis exclusion. To block glycolysis and oxidative phosphorylation (OXPHOS) with oxygen available, the authors used iodoacetic acid (100 μM) and oligomycin A (5 μg/ml), respectively; under anoxic conditions with no electron acceptor on ETC complex IV, the addition of the F1–F0–ATP synthase inhibitor was excluded (Figure 2 (a)). The period of the cell ability to maintain the difference in Ca^2+^ concentrations relative to the cytoplasmic membrane, characterizing the consumption of available ATP reserves, turned out to be statistically significantly lower in case of hypoxia compared to the experiment with blocking glycolysis and OXPHOS (3.1 [2.8; 3.3] and 4.4 [4.2; 4.6] h, respectively; p<0.0001; N=3; n=33 cells) (Figure 2 (b)). This indicated the efficiency of physical oxygen removal and even the activation of additional energy-consuming processes. Considering the mitochondrial consumption of over 95% of the oxygen consumed by cells [46], the difference between the two experiments can be explained by a change in the state of these organelles. As it is clear from the confocal images (Figure 2 (c)–(e)), the TMRM fluorescence intensity in the mitochondria of cells incubated in the presence of Na_2_S_2_O_4_ for 30 min results in a significant decrease in ΔΨm. If, in case of complete blocking of ATP synthesis, the ΔΨm level in cells was 108.1 [82.8; 139.8]% of the control, then with oligomycin A replacement with Na_2_S_2_O_4_, the same level was sharply decreased (10.7 [9.7; 11.9]% of the control value; N=3; n=40 cells) (Figure 2 (f)). At that, as one can see in Figure 2 (i), under hypoxic conditions, depolarization of mitochondria is observed after oligomycin A application, which indicates a disruption in the ETC complex V functioning.

*Oxygen binding by Na_2_S_2_O_4_ triggers cell death due to apoptosis.*

**Figure 3.**
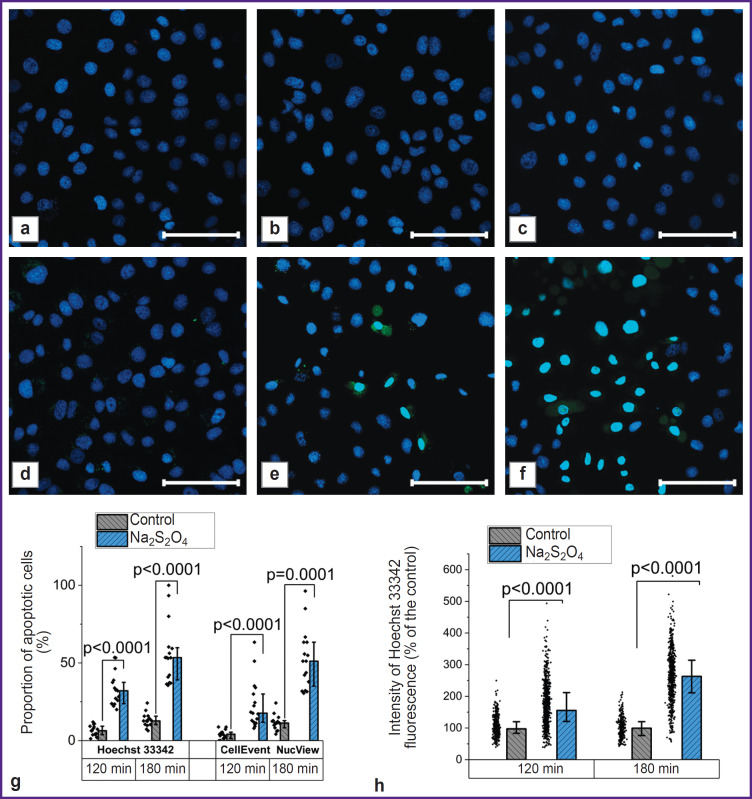
In model hypoxia, cell death occurs due to apoptosis, which can be identified using different methods Confocal images of cells loaded with Hoechst 33342 and propidium iodide under control (a) and hypoxia conditions after 2 h (b) and 3 h (c), as well as Hoechst 33342 and NucView under control conditions (d) and under hypoxia conditions after 2 h (e) and 3 h (f); results of calculated proportion of apoptotic cells using two methods (g); results of the level of cell apoptosis assessment based on the fluorescence intensity of Hoechst 33342 (h). Bar is 100 μM

Depletion of ATP reserves ultimately led to cell death, which could occur due to necrosis or apoptosis. The analysis using Hoechst 33342, which stained the nuclei of all cells, and propidium iodide, which penetrated only through damaged membranes, demonstrated that the progression of necrosis was not typical for the studied model conditions (the proportion of cells stained with propidium iodide did not exceed 0.1% in both the control culture and the culture that was treated with Na_2_S_2_O_4_ for 2 and 3 h) (Figure 3 (a)–(c)).

In order to assess the progression of alternative cell death pathways, the authors conducted examinations using the NucView selective substrate, which acquired the ability to fluoresce after enzymatic hydrolysis and stained the nuclei of apoptotic cells. In case of hypoxia, a significant increase in the number of cells with signs of apoptosis was observed (Figure 3 (d)–(f)). Similar conclusions can be made based on a more detailed study of the results of the nuclei staining with the fluorescent probe Hoechst 33342. The use of this probe to assess the progression of apoptosis was due to the ongoing changes in the nuclei morphology, as well as the fluorescence intensity [47]. A comparative analysis of the calculated proportion of apoptotic cells using two fluorescent probes showed similar results when using the NucView selective substrate and Hoechst 33342 (Figure 3 (g)). At that, in the second case, a transition to a more objective assessment based on determining the fluorescence intensity of the probe was possible (Figure 3 (h)): after 120 min of hypoxia, this parameter amounted to 155.3 [120.7; 211.8]% of the control value (p<0.0001; N=3; n=484 cells), and after 180 min — 263.0 [211.0; 313.9]% of the control value (p<0.0001; N=3; n=392 cells).

*The use of Na-fumarate increases the cells viability by reducing the rate of apoptosis progression in case of hypoxia.*

**Figure 4.**
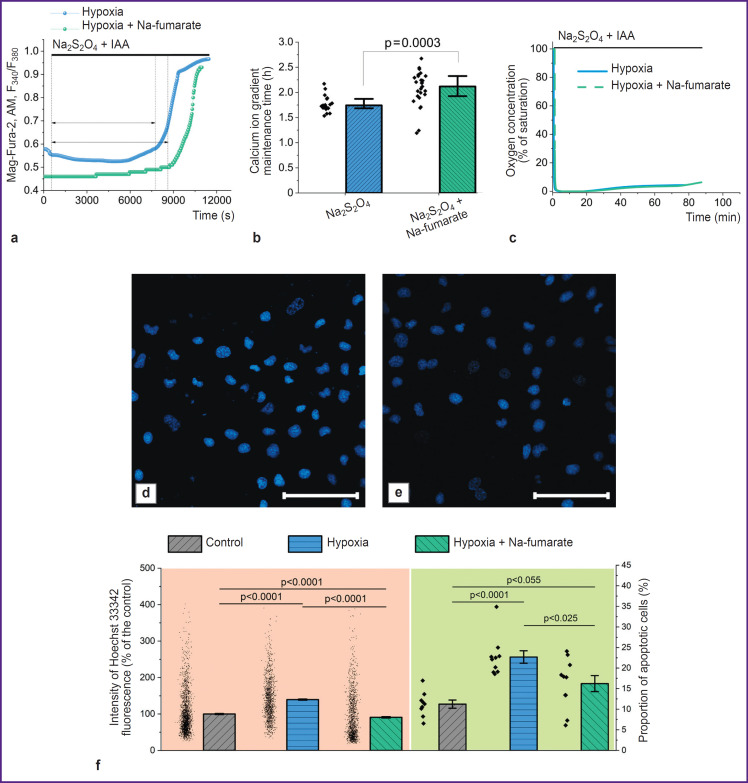
Use of the hypoxia model allows identifying the protective impact of antihypoxants exemplified by a Nafumarate solution Plots of changes in the cytosolic concentration of Ca^2+^ and Mg^2+^ (a) and results of statistical analysis (b) of the duration of maintenance of the calcium ion concentration gradient by cells relative to the cytoplasmic membrane; plots of polarographic determination of the oxygen concentration in the medium (c); confocal images of cells stained with Hoechst 33342 in case of hypoxia (d) and with the addition of Na-fumarate (e); the level of apoptosis in cells is determined by changes in the morphology and fluorescence intensity of cell nuclei stained with Hoechst 33342 (f). Bar is 100 μm

Despite the use of Na-fumarate in clinical practice, the mechanism of its action in case of acute hypoxia seems to be poorly understood. Thus, the authors used a model of acute hypoxia based on Na_2_S_2_O_4_ to observe the changes occurring at the cellular level (Figure 4). Incubation of MDCK cells under hypoxic conditions with 5 mM Na-fumarate caused an increase in the period of the ability to maintain the difference in Ca^2+^ concentration relative to the cytoplasmic membrane compared to the control experiment without a nephroprotector (Figure 4 (a)) by 26.6% (p=0.032; N=3; n=17 cells) (Figure 4 (b)). This effect was not related to the hypothetical ability of Na-fumarate to oxidize Na_2_S_2_O_4_, hence reducing the duration of hypoxia (Figure 4 (c)). Here the authors noted significantly smaller changes in the morphology of nuclei and an increase in the fluorescence intensity of Hoechst 33342 (Figure 4 (d), (e)), which indicated a decrease in the rate of pathological processes leading to apoptosis. Statistical analysis showed (Figure 4 (f)) that the proportion of apoptotic cells in experiments with 3-hour hypoxia and with the addition of Nafumarate was significantly lower than in experiments without it (p<0.025; N=3; n=15 fields of view). Moreover, a similar result was seen when assessing apoptosis by the increase in the fluorescence intensity of Hoechst 33342 associated with chromatin condensation. In case of hypoxia without additional exposure, the fluorescence intensity of the probe was 131.5 [99.8; 170.0]% of the control value (p<0.0001; N=3; n=1278 cells), with Na-fumarate availability this parameter was reduced to 68.3 [42.8; 127.7]% of the control value (p=0.055; N=3; n=1326 cells).

*Na-fumarate ensures partial oxidation of mitochondrial coenzymes with preservation of the inverse mode of ETC complex V.*

**Figure 5.**
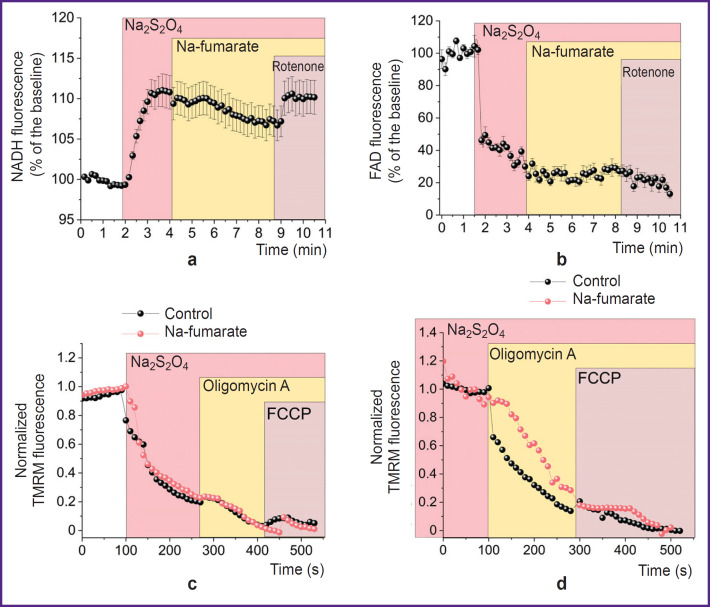
Functioning of individual complexes of the mitochondrial electron transport chain with Na-fumarate in case of hypoxia Plots of changes in cell autofluorescence when exposed to exciting radiation with a wavelength of 360 nm (a) and 450 nm (b) after creating hypoxia and introducing Na-fumarate; plots reflecting the mechanism for ΔΨm maintaining in case of hypoxia with short-term (c) and long-term (d) incubation of cells with Na_2_S_2_O_4_ and Na-fumarate

The increase in the period of ability to maintain the difference in Ca^2+^ concentration relative to the cytoplasmic membrane under fumarate (see Figure 4 (a)) indirectly showed a higher level of ATP in the cells, although the corresponding mechanism remains unclear. The possibility of regular functioning of the ETC and the occurrence of OXPHOS in the simulated conditions could be tested by studying the state of mitochondrial coenzymes by the level of cell autofluorescence upon excitation at a wavelength of 360 nm (for NADH) (Figure 5 (a)) and 450 nm (for FAD) (Figure 5 (b)). In both cases, after recording the baseline autofluorescence level, a sharp change in the signal was observed upon addition of Na_2_S_2_O_4_, which was explained by the complete transition of the coenzymes in the mitochondria into reduced forms — NADH and FADH_2_, the latter of which could not fluoresce. The subsequent addition of Nafumarate to a working concentration of 5 mM resulted in an insignificant decrease in NADH fluorescence (see Figure 5 (a)) and an increase in FAD (see Figure 5 (b)). This indicated their oxidation by complexes I and II, respectively. The said changes were not immediate (they were seen approximately after 2 min) after the introduction of fumarate, which was probably associated with the processes of transmembrane transport of the substance. The subsequent reverse changes under the influence of rotenone (2 μM) confirmed the impact of the NADH dehydrogenase complex associated with Nafumarate. However, according to the results of the ΔΨm formation mechanism examination, in case of both short-term (about 3 min) (Figure 5 (c)) and longterm (30 min) (Figure 5 (d)) incubation of cells under hypoxic conditions with Na-fumarate, depolarization of the inner mitochondrial membrane in response to oligomycin A was maintained, thus proving a disruption in F1–F0–ATP synthase function.

*Incubation of cells with Na-fumarate under normoxic conditions increases the ability of cells to uptake glucose and acidify the cytosol.*

**Figure 6.**
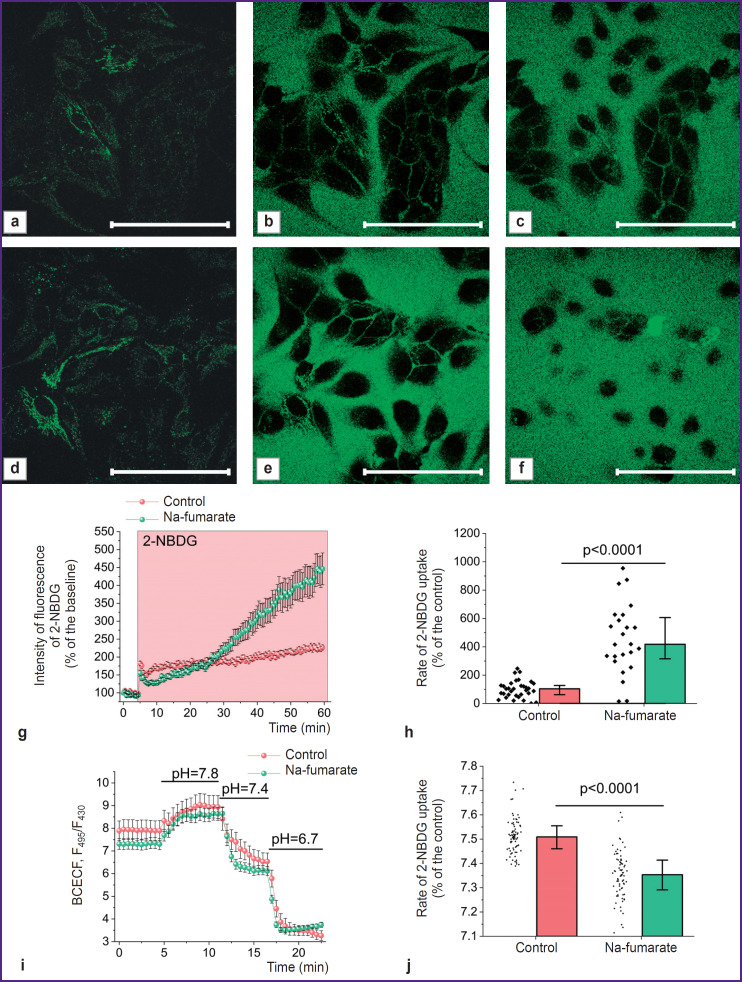
Changes in cells affected by Na-fumarate and associated with HIF1α accumulation at normal dissolved oxygen level Microphotographs of MDCK cells with fluorescence excitation by a 488 nm laser before (a), (d), at (b), (e), and 60 min after (c), (f) introduction of 2-NBDG for the control culture (a)–(c) and cultures incubated with Na-fumarate for 24 h (d)–(f); averaged plots of fluorescence signal changes with excitation by a 488 nm laser in experiments on 2-NBDG absorption by control cells and cells incubated with Na-fumarate (g); results of statistical analysis of the impact of cells incubation with Na-fumarate on the rate of 2-NBDG absorption (h); curves of F_495_/F_430_ changes in the study of fluorescence of the pHdependent BCECF probe with changes in the pH of the medium in control cells and incubated with Na-fumarate for 24 h (i); results of statistical analysis of the impact of Na-fumarate on cytosolic pH (j). Bar is 100 μm

As the use of Na-fumarate in kidney surgery involves intravenous administration of the medication for several days before surgery [6], the authors assessed the possibility of increasing the role of alternative to OXPHOS pathways of ATP synthesis before the onset of hypoxia. Thus, there were experiments performed in which cell cultures were incubated for 24 h in a complete growth medium containing 5 mM Na-fumarate, followed by an analysis of the rate of glucose uptake and the pH level of the cytosol. The experiments using the fluorescent probe 2-NBDG (Figure 6 (a)–(h)) established that the control and treated cultures differ significantly in the rate of glucose transport through the plasma membrane, which may indicate a significantly higher level of expression of the glucose transporter 1 gene (*GLUT1*). Statistical analysis demonstrated that daily incubation with Na-fumarate resulted in a more than 4-fold increase in the rate of 2-NBDG absorption (p<0.0001) (see Figure 6 (g), (h)).

To assess the intracellular pH level, the authors used the ratiometric probe BCECF, the fluorescence intensity ratio of which greatly depended on the acidity index (Figure 6 (i), (j)). The presented data suggest that (see Figure 6 (j)), even under normoxia, incubation of cells with Na-fumarate results in a statistically significant decrease in pH — from 7.51 [7.46; 7.55] for the control value to 7.35 [7.29; 7.41] in case of the experimental culture (p<0.0001; N=3; n=79 cells).

## Discussion

The practical application of Na-fumarate in kidney resection includes its infusion before the surgery in order to increase its content in the body tissues. At that, morphological and clinical-biochemistry studies indicate a nephroprotective function of Na-fumarate when using the technique of warm ischemia of the organ [6]. However, the corresponding changes in cellular metabolism and the mechanism of cell protection are understudied to date [18–20, 48]. Moreover, there is still a question whether these changes are associated with the action of the substance during the period of direct oxygen removal or are mediated by preliminary activation of protective mechanisms. It would be safe to assume that answers to these questions can be found in *in vitro* studies.

To simulate acute hypoxia in experiments on MDCK cell culture, oxygen reduction using Na_2_S_2_O_4_ was used. Despite the advantages related to an almost instantaneous decrease in the dissolved oxygen content to a level of 1–2% of saturation (see Figure 1 (a)), this approach requires clarification and optimization for specific experimental conditions. For instance, a sufficiently high reactivity and, thus, low stability bring up the issue of the time during which the compound will ensure hypoxia. The impact of a single introduction of Na_2_S_2_O_4_ at a concentration of 5 mM depends on the thickness of the medium layer above the cells (see Figure 1 (b)) and is limited to a period of up to 120 min, which, with a longer incubation, requires a new introduction of the reducing agent or, preferably, to exclusion of its contact with atmospheric air.

Acute hypoxia leads to significant changes in mitochondrial metabolism. Blocking the electron transport in the ETC with no terminal electron acceptor and, thus, blocking the function of complexes I, III, and IV that transfer protons into the transmembrane space, resulted in a significant depolarization of mitochondria, which was enhanced by oligomycin A introduction (see Figure 2 (i)). This indicates the transition of complex V to ATPase mode in order to maintain ΔΨm, which is typical for many pathologies associated with mitochondrial dysfunction [49]. Here, disturbances in cellular metabolism in the model conditions lead to cell apoptosis. This process can be assessed using methods specific to a particular mechanism of cell death (for example, the NucView fluorescent substrate application) or based on a non-specific assessment of changes in both the nuclei morphology and the fluorescence intensity of the Hoechst 33342 chromatin-binding [50, 51]. Considering that apoptosis progression mechanisms can be both caspase-dependent and caspaseindependent [47], the latter approach is promising for *in vitro* screening studies of medications developed and recommended for use in simulated organ ischemia.

The results of assessing apoptosis and the cells ability to facilitate energy-consuming processes indicate that fumarate has pronounced protective properties under hypoxic conditions (see Figure 4 (f)), associated with an increase in the ATP level. Possible explanations for the formation of ATP in mitochondria under anaerobic conditions include, for example, substrate phosphorylation resulting from the conversion of α-ketoglutarate to succinate [18, 19], or the reverse action of succinate dehydrogenase, which reduces fumarate to succinate, thus allowing complex I to oxidize NADH and transfer protons into the transmembrane space with their subsequent return via F1–F0–ATP synthase and the ATP formation [18–20]. On the other hand, there are reasons for the extremely low probability of the latter pathway [50]. The data obtained provide that addition of Na-fumarate leads to a change in the NADH/NAD and FADH_2_/FAD ratios, which are seen due to a decrease and increase in the autofluorescence level, respectively (see Figure 5 (a), (b)). This indicates a partial restoration of the ETC complex I function due to conversion of fumarate to succinate under succinate dehydrogenase action. However, complex V does not enable the F1–F0–ATP synthase mode: the TMRM fluorescence intensity decreases after the oligomycin A addition (see Figure 5 (c), (d)), which indicates the preservation of the leading role of ATPase mode of complex V operation in ΔΨm maintenance. Thus, the possible partial restoration of complex I function is not associated with proton transfer through the inner mitochondrial membrane or may be insufficient to form the ΔΨm level required for OXPHOS [48]. Hence, substrate phosphorylation in the mitochondrial matrix [18, 19], even if it occurs under the model conditions, will ensure the ATP formation for complex V functioning, which does not explain the increase in cell viability.

The antihypoxic effect of Krebs cycle substrates may be related to the activation of intracellular processes associated with HIF1α, which is unstable under aerobic conditions and is a central link in the hypoxia-induced state progression and together with HIF1β is involved in the HIF1 heterodimer formation [52]. This assumption is based on the data on inhibition of HIF1α-oxidizing prolyl hydroxylases by fumarate or succinate in cancer cells with a deficiency of fumarate hydratase and/or succinate dehydrogenase, which results in pseudohypoxia even with normal oxygen content in the medium [21–23]. This leads to an increase in glucose consumption by cells due to an increase in *GLUT1* gene expression, as well as to glycolysis contributing to ATP production (hexokinase 2 and pyruvate kinase genes). Despite the lower yield of high-energy compounds (conversion of one glucose molecule results in formation of two ATP molecules), glycolysis can meet the energy needs of cells due to its relative simplicity and higher rate [53].

Accumulation of HIF1α under increasing fumarate levels may also be associated with phosphorylation of the p65 protein and activation of the NF-κB pathway [24]. Moreover, inhibition of enzymes from the TET family by fumarate or succinate results in a change in the expression level of HIF-regulated genes [25]. Finally, there is evidence of a possible interaction of fumarate accumulating in cells accompanied with fumarate hydratase deficiency with the KEAP1 protein, which leads to activation of the NRF2 transcription factor [26]. In general, the possibility of using a protective mechanism related to stabilization of HIF1α during hypoxia was considered in many studies [27, 28], but it is unknown whether the protective effect of exogenous fumarate can be associated with this possibility.

In case of kidney protection during warm ischemia, the maximum cytoprotective effect is most often achieved by starting protective activities before the surgical procedure. Here, the authors conducted studies with 24-hour incubation of cells with Na-fumarate at a normal oxygen level, which revealed a significant increase in the rate of glucose consumption by cells (see Figure 6 (h)), as well as a statistically significant decrease in the pH level of cytosol (Figure 6 (j)). Cumulatively, this may indicate a switch in metabolism to anaerobic glycolysis, which is typical of anaerobic conditions, the end product of which is lactate [54]. The progression thereof is due to activation of protective mechanisms, the most important element of which is the HIF1α stabilization [48, 49].

## Conclusion

Possible risks of limiting the blood supply to the surgically treated organs and tissues can be reduced by using antihypoxants, in particular Na-fumarate, and understanding the mechanism of action of such compounds will allow them to be used more effectively in practice. *In vitro* studies established that with a sharp decrease in oxygen in the medium simulating acute hypoxia conditions, the cells not significantly lose the ability to implement normal energy-consuming processes, but also undergo transition of the mitochondrial ETC complex V to ATPase mode, which leads to cell death mainly due to apoptosis. At that, in case of hypoxia Na-fumarate demonstrates its ability to reduce the level of cell apoptosis. This is due to an increase in the ATP content in cells, which, however, is not ensured by the restoration of mitochondrial function. Incubation of cells with Na-fumarate under normoxic conditions results in an increase in the rate of glucose consumption by cells, as well as in a significant decrease in cytosolic pH, which may be a consequence of a higher level of expression of glucose transporter genes and key glycolytic enzymes regulated by HIF1α, as well as may explain the demonstrated cytoprotective effect.
